# Halting targeted and collateral damage to red blood cells by the complement system

**DOI:** 10.1007/s00281-021-00859-8

**Published:** 2021-06-30

**Authors:** M. Jalink, E. C. W. de Boer, D. Evers, M. Q. Havinga, J. M. I. Vos, S. Zeerleder, M. de Haas, I. Jongerius

**Affiliations:** 1 Sanquin Research, Center for Clinical Transfusion Research, Plesmanlaan 125, 1066 CX Amsterdam, The Netherlands; 2 Department of Hematology, Amsterdam UMC, University of Amsterdam, Amsterdam, The Netherlands; 3 Sanquin Research, Department of Immunopathology, and Landsteiner Laboratory, Amsterdam University Medical Centre, Amsterdam Infection and Immunity Institute, Amsterdam, The Netherlands; 4 Department of Pediatric Immunology, Rheumatology, and Infectious Diseases, Emma Children’s Hospital, Amsterdam University Medical Centre, Amsterdam, The Netherlands; 5 Department of Hematology, Radboud University Medical Center, Nijmegen, The Netherlands; 6 Lymphoma and Myeloma Center Amsterdam (LYMMCARE), Amsterdam, The Netherlands; 7 Department of Immunohematology Diagnostics, Sanquin, Amsterdam, The Netherlands; 8 Department of Hematology and Central Hematology Laboratory, Inselspital, Bern University Hospital, University of Bern, Bern, Switzerland; 9 Department for BioMedical Research, University of Bern, Bern, Switzerland; 10 Department of Hematology, Leiden University Medical Center, Leiden, The Netherlands

**Keywords:** Autoimmune hemolytic anemia, Paroxysmal nocturnal hemoglobinuria, Complement, Complement inhibitors, Complement therapeutics

## Abstract

The complement system is an important defense mechanism against pathogens; however, in certain pathologies, the system also attacks human cells, such as red blood cells (RBCs). In paroxysmal nocturnal hemoglobinuria (PNH), RBCs lack certain complement regulators which sensitize them to complement-mediated lysis, while in autoimmune hemolytic anemia (AIHA), antibodies against RBCs may initiate complement-mediated hemolysis. In recent years, complement inhibition has improved treatment prospects for these patients, with eculizumab now the standard of care for PNH patients. Current complement inhibitors are however not sufficient for all patients, and they come with high costs, patient burden, and increased infection risk. This review gives an overview of the underlying pathophysiology of complement-mediated hemolysis in PNH and AIHA, the role of therapeutic complement inhibition nowadays, and the high number of complement inhibitors currently under investigation, as for almost every complement protein, an inhibitor is being developed. The focus lies with novel therapeutics that inhibit complement activity specifically in the pathway that causes pathology or those that reduce costs or patient burden through novel administration routes.

## Introduction

The complement system is part of innate immunity, and abnormalities in its regulation have been associated with a wide range of pathologies [1]. Red blood cells (RBCs) seem particularly sensitive to dysregulation of the complement system, which is not surprising as RBCs are continuously exposed to complement components [2]. Either an intrinsic deficiency in complement regulation on RBCs or an extrinsic excessive complement activation against RBCs can induce premature and sometimes fulminant destruction of these cells, of which paroxysmal nocturnal hemoglobinuria (PNH) and autoimmune hemolytic anemia (AIHA), respectively, are highly characteristic. Eculizumab was the first complement inhibitor to be approved for clinical use and has revolutionized the treatment of PNH. Yet many challenges remain, including the lack of any approved complement inhibitors for the treatment of AIHA. Novel complement inhibitors to improve the treatment of PNH and address complement-mediated AIHA are currently being developed [3–5].

This review aims to give an overview of developments within the field of complement-targeting therapeutics that may in the future further optimize treatment and outcomes of complement-mediated hemolytic diseases. To this end, the working mechanism of the complement system and its contribution to the pathology of PNH and AIHA are first discussed. Second, current available complement-regulating agents and novel therapeutic developments are discussed, including potential advances in novel targets, efficacy, side effects, administration route, and patient burden.

## The complement system

The complement system is an important part of innate immunity. The system is composed of plasma proteins that activate one another in a cascade. Due to its continuous presence in plasma, the system is readily available and can quickly respond to triggers, supporting the elimination of bacteria, apoptotic cells, and immune complexes. These characteristics give the system a key role in the defense against pathogens, but it also plays a role in tissue homeostasis [6–9]. In addition to its role in the innate immune system, the complement system also has a modulating role in the adaptive immune system [10].

**Fig. 1:**
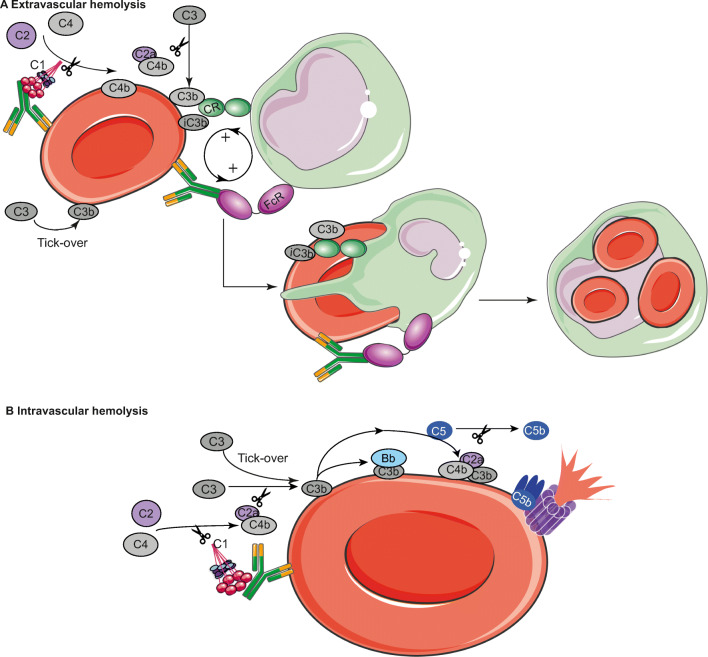
Mechanisms of extravascular and intravascular hemolysis. (**A**) Complement activation on RBCs can occur via the CP (in AIHA) or via the AP (in PNH). Extravascular hemolysis via the CP is the consequence of opsonization of the RBC with antibodies, fragments of C4 (C4b or C4d), and/or fragments of C3 (C3b, iC3b, or C3d). Extravascular hemolysis via the AP (tick-over) depends on opsonization with C3 fragments only. Phagocytes express Fc and complement receptors, which bind to antibodies or the complement components on the target cell, respectively. The synergy between both receptors results in highly effective phagocytosis. Upon phagocytosis, the whole RBC is internalized into the phagocyte within the phagosome. (**B**) Intravascular hemolysis can be initiated by both the AP and CP. C1q can bind to antibodies on an RBC, which induces the cleavage of C2 and C4, forming the CP convertase C4b2a. Both the CP convertase and spontaneous background hydrolysis (tick-over) of C3 in the AP result in C3b cleavage. C3b can then deposit on the cell, or bind to C4bC2a, forming the C5 convertase. Upon cleavage of C5, C5b is formed, which associates with C6, C7, C8, and multiple C9 molecules to form the membrane attack complex (MAC), which inserts itself into the cell membrane, resulting in lysis. Figure created using Servier Medical Art

The activation of the complement system can occur via three different pathways: the classical, lectin, and alternative pathways. These pathways each have their specific recognition molecules with corresponding triggers (reviewed in previous studies [7, 8]). In brief, the classical pathway (CP) is initiated by C1q, recognizing antibodies bound to target cells, activating C1r which in turn activates the serine protease C1s and its downstream pathway [8, 11]. The lectin pathway (LP) is activated via mannose-binding lectin (MBL), collectins, and ficolin which recognize microbial carbohydrate structures. Upon recognition of their specific patterns, they form a complex with MBL-activated serine proteases (MASPs) which induces further activation of the LP [12]. Both CP and LP activation result in C4 and C2 cleavages, which leads to the formation of the C3 convertase (C4bC2a) that can cleave C3 into C3a and C3b [8]. Lastly, the alternative pathway (AP) can be activated spontaneously by background hydrolysis of C3, and it acts as an amplification route of complement activation, as it is activated following C3b deposition via the other pathways. Factor B (FB) will bind to C3b and upon cleavage by factor D (FD); the C3 convertase (C3bBb) is formed. Similar to the C3 convertase of the CP/LP, this convertase cleaves C3, forming C3a and C3b [8, 13]. Both C4b and C3b, formed upon complement activation, opsonize target cells, which induces phagocytosis. Furthermore, C3b also contributes to the formation of C5 convertases, which cleaves C5 into C5a and C5b. C5b interacts with C6, resulting in subsequent binding of C7, C8, and multiple C9 molecules. These molecules together form the membrane attack complex (MAC) that creates a pore by inserting into the membrane of the target cell, resulting in cell lysis [8]. In the context of RBCs, opsonization and subsequent phagocytosis is a process of extravascular hemolysis (Fig. 1A), while MAC activation and subsequent cell lysis refer to intravascular hemolysis (Fig. 1B) [2]. Cleavage of C3 and C5 also results in the release of the anaphylatoxins C3a and C5a, which are chemoattractants and modulators of inflammation. For example, they recruit macrophages and neutrophils and induce pro-inflammatory cytokine production by T cells and antigen-presenting cells [9, 14].

As the complement system can be very harmful and is continuously ready to be activated by invading foreign organisms, it is important to prevent unwanted complement attacks of host cells. In order to protect host cells, an intricate system of complement regulators is in place to keep the complement system under control. Regulators can effectively modulate all steps in the complement activation cascade and can be divided into two groups: the membrane-bound and the soluble complement regulators in plasma [6]. The membrane-bound complement regulators consist of complement receptor 1 (CR1 or CD35), membrane co-factor protein (MCP or CD46), decay-accelerating factor (DAF or CD55), complement receptor of the immunoglobulin family (CRIg), and CD59 (Table 1). CR1 binds C3b and C4b and can induce the decay of both C3 and C5 convertases but also functions as a co-factor for factor I (FI) [21, 22]. CD46 can also bind C3b and C4b but does not have any decay-accelerating activity in itself, realizing its regulatory activity solely as an FI co-factor [23, 24]. CD55 and CD59 are both glycosylphosphatidylinositol (GPI)-anchored membrane-bound complement regulators. CD55 accelerates the decay of the C3 convertases of all pathways, while CD59 acts on the terminal pathway by preventing MAC formation [6, 25]. Lastly, CRIg binds to C3b and iC3b and inhibits the AP C3 convertase [18, 26]. The expression profile of these membrane-bound regulators differs per cell type. For example, RBCs, the main subject of this review, have CR1, CD55, and CD59 on their surface but lack CD46 and CRIg [24].

**Table 1:** Complement receptors and their respective ligands

Receptor	Ligands	Expressed on	Effect	Ref.
CR1 or CD35	C3b, C4b	RBCs, monocytes, macrophages, renal podocytes	Complement regulation by decay of C3 and C5 convertases, induction of phagocytosis	[8, 10, 15]
CR2 or CD21	iC3b, C3d, C3dg	B cells	Co-stimulatory in B cell activation	[8, 10, 15, 16]
CR3 or CD11b/CD18	C3b, iC3b, C3d	Leucocytes	Induction of phagocytosis	[8, 10, 15, 17]
CR4 or CD11c/CD18	C3b, iC3b, C3c	Leucocytes	Induction of phagocytosis	[8, 10, 15, 17]
CRIg	C3, iC3b, C3c	Dendritic cells, Kupffer cells	Induction of phagocytosis, C3 convertase inhibition	[10, 15, 18–20]

The group of soluble complement regulators consists of C1 inhibitor (C1-INH), C4 binding protein (C4BP), factor H (FH), clusterin (CLU), vitronectin (Vn), and FI which are constitutively present in plasma. C1-INH and C4BP regulate both the LP and CP [27, 28]. FH on the other hand is a strong regulator of the AP, with recognition sites for host cells and C3b in order to prevent or reverse the formation of the AP convertase on host cells [29, 30]. Both CLU and Vn inhibit MAC formation [6, 28]. Lastly, FI inactivates C3b and C4b and thus regulates all complement pathways. For this, FI needs the aid of a co-factor, a role that is played by other complement regulators: C4BP, FH, CR1, or CD46 [31]. The inactivation of C3b by FI is mediated by the subsequent cleavage of C3b to iC3b and of iC3b to C3c and C3dg. For the latter, only CR1 can act as a co-factor, and this step is key to prevent iC3b-mediated activation of neutrophils through CR3 binding, which is described subsequently in more detail [32]. The sensitivity of RBCs to complement-mediated attack depends on the protection offered by both membrane-bound and soluble complement regulators [25].

As described previously, complement activation leads to opsonization of target cells with C3b and C4b, which is of major importance for phagocytosis. CRs are membrane-bound molecules expressed on various immune cells and play a key role in this phagocytic process (see Table 1). Recognition of C3b and C4b by CRs will lead to activation of immune cells, resulting in induction of phagocytosis. Currently, five complement receptors have been identified: CR1, CR2, CR3, CR4, and CRIg [8]. Next to its role in complement regulation, CR1 is expressed on RBCs, monocytes, macrophages, neutrophils, and renal podocytes and can bind both C3b and C4b. CR2 is structurally similar to CR1, although it is only expressed on B cells and thus mainly serves as a co-stimulatory molecule for activation of the B cell upon the interaction between antigen and B cell receptor, in which case CR2 amplifies signal transduction [8, 15, 16, 33]. This could play a role in AIHA, where C3d is detected on the RBC surface, as this could thus induce increased B cell receptor signaling and subsequent antibody production [34]. CR3 and CR4 both belong to the integrin family but recognize different C3 fragments (see Table 1). They are expressed on leukocytes, especially on macrophages, monocytes, and NK cells, but also on some B and T cells [17]. Lastly, CRIg is the most recently discovered complement receptor, which belongs to the immunoglobin superfamily. CRIg is expressed only on dendritic cells and Kupffer cells, which are subtypes of macrophages specifically present in the liver [19]. Kupffer cells play an important role in the clearance of pathogens from the bloodstream by phagocytosis, and their contribution to intravascular hemolysis has not been elucidated yet. For the binding of C3 fragments and clearance of C3-opsonized pathogens by Kupffer cells, CRIg has been deemed essential based on studies in knockout mice [26].

Apart from complement-mediated phagocytosis, IgG-Fc receptors (FcR) on phagocytes can initiate antibody-mediated phagocytosis, which is of major importance in AIHA, as described subsequently. These receptors bind to the Fc region of antibodies that have opsonized cells or other particles. FcRs come in different types, which differ in the antibody (sub)classes they recognize and their affinity for the antibodies. Next, they can carry out specific immune effector functions [35–37]. Antibody characteristics, such as antibody class and Fc-glycosylation profile, are important for both interactions between antibody and FcR and complement activation [35, 36, 38]. Splenic red pulp macrophages, involved in clearing IgG-opsonized RBCs, distinctively express all types of activating FcγRs and are specifically important in the pathophysiology of AIHA [39]. Altogether, the interplay between complement and antibody opsonization of target cells and subsequent activation of CRs and FcRs determines the process of phagocytosis and is of synergistic nature, which, in the context of RBCs, results in extravascular hemolysis (Fig. 1A). C3 fragment opsonization seems mainly responsible for particle binding by phagocytes, while IgG opsonization seems important for particle ingestion [40]. The presence of C3 fragments together with IgG on a particle can significantly reduce the amount of IgG required to induce ingestion and even seems essential for effective induction of phagocytosis [41].

## Complement-mediated diseases affecting RBCs

Several pathologies have been described where overactivation and dysregulation of the complement system induce RBC damage. This is not surprising, as RBCs are continuously exposed to the complement system in the bloodstream [2]. Of these pathologies, PNH, AIHA, and aHUS are best defined but in more rare cases involving deficiencies of a single complement regulator (CD55 or CD59) causing similar symptoms have been described [42]. In this review, we will focus on the underlying mechanisms and clinical management of AIHA and PNH, as the damage to RBCs in these pathologies is fully complement mediated. The pathology of aHUS has been extensively reviewed [43], and the common belief is that hemolysis in aHUS is not solely complement mediated but merely a result of mechanistic hemolysis, which falls outside the scope of this current review [43]. However, very recent data obtained in an in vitro model of aHUS showed that hemolysis can be a direct result of AP activity as well [44]. More research will be needed to determine the role of the complement system on RBC destruction in aHUS in vivo*.*

### Complement in PNH

**Fig 2.**
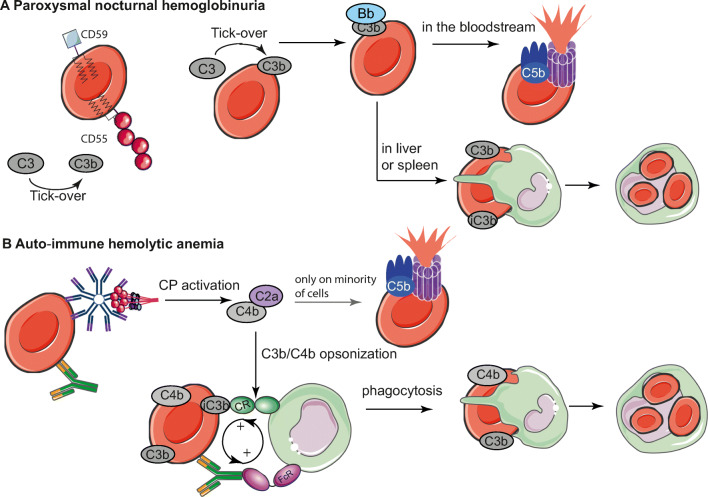
Mechanism behind complement-mediated destruction of RBCs in PNH and AIHA. (**A**) Healthy RBCs express the GPI-anchored complement regulator CD55, which induces decay of C3 convertases, and CD59, which prevents MAC formation. PNH RBCs, however, do not express these regulators, meaning that C3b arising from tick-over can result in opsonization of the RBC. On PNH RBCs, further complement activation is not prevented and can result in MAC formation and intravascular hemolysis or in extravascular hemolysis by phagocytosis of C3 fragment-opsonized RBCs in the liver or spleen, which are often iC3b or C3b opsonized in PNH patients. (**B**) RBC autoantibodies in AIHA patients of either IgG or IgM class bind to RBCs and can induce CP activation, which leads to further opsonization of the RBC with complement and in some cases to MAC formation and direct lysis. The opsonized RBC can be phagocytosed via the IgG-Fc receptors and complement receptors. Figure created using Servier Medical Art

PNH is a rare hemolytic disease with an estimated incidence of 1–1.5 cases per million individuals worldwide [45]. Clinical disease is caused by clonal expansion of hematopoietic stem cells with an acquired somatic mutation in the phosphatidylinositol glycan-A (PIGA) gene. The absence of PIGA enzyme activity results in hematopoietic cell deficiency in GPI-anchored proteins, including the complement regulators CD55 and CD59. As CD55 is highly important in regulating the AP and C3 convertases, while CD59 prevents the formation of the MAC, affected cells are rendered highly vulnerable to the effects of activation of C3, C5, and the terminal pathway of complement, culminating in the formation of the MAC (Fig. 2A) [46]. Protection of PNH RBCs against AP activity by the soluble complement regulator FH is very important and does allow the RBCs to endure in circulation for days instead of immediate cell lysis, which might be expected in cells deficient in GPI-anchored proteins [47].

Hemolytic anemia, mainly due to intravascular hemolysis, is the hallmark of PNH, resulting in the release and accumulation of free hemoglobin and iron in plasma, subsequent nitric oxide (NO) depletion, and upregulation of pro-inflammatory cytokines. Thrombosis, at any site and both venous and arterial, has been the major direct cause of death in PNH prior to the availability of terminal complement inhibitors. Various mechanisms play a part here, all characterized by excessive complement activation against hematopoietic cells, including the formation of prothrombotic platelet microvesicles, platelet activation, and platelet aggregation via NO scavenging. There is defective fibrinolysis related to a lack of several GPI-linked coagulation regulators and a general pro-inflammatory state. Several of its other symptoms, such as dysphagia and erectile dysfunction, are related to general smooth muscle dystonia as a consequence of the overall NO scavenging [3, 45, 48].

### Current management of PNH

The treatment of PNH has drastically improved since the introduction of terminal complement inhibitors [49]. Eculizumab is a recombinant humanized monoclonal antibody that selectively targets C5, preventing its cleavage into C5a and C5b and subsequently the formation of the MAC [49, 50]. As such, it compensates for the CD59 deficiency in PNH but does not overcome the CD55 deficiency. Indeed, two large phase 3 clinical studies (TRIUMPH study [NCT00122330] and SHEPHERD study [NCT00130000]) allow for a significant reduction of intravascular hemolysis, transfusion requirements, and thrombosis incidences following initiation of eculizumab treatment [51, 52]. In almost all patients, a mild to severe residual anemia persists, with about 30% of patients remaining transfusion dependent [46, 53]. Next to underlying PNH-related bone marrow failure, transfusion dependency is explained by extravascular hemolysis due to ongoing C3b deposition on RBCs [51, 52].

Further developments of eculizumab analogs with modifications of its Fc regions resulted in enhanced recycling [54]. This new C5-specific monoclonal antibody, ravulizumab, only very recently proved equally efficient as compared to eculizumab, benefits from an approximately fourfold increased half-life, and thereby a dosing interval of 8 rather than 2 weeks as compared to eculizumab [55].

Although eculizumab is effective in most patients, failure to respond to eculizumab has been described. This is mostly observed among Asian PNH patients bearing a C5 polymorphism, with a prevalence of approximately 3.2% among Japanese and 1% among Chinese Han patients with PNH [56]. This genetic variant of C5 prevents binding of eculizumab and thus abrogates adequate complement inhibition. Another C5 variant interfering with the binding of eculizumab was recently described in a Caucasian male patient. Upon treatment switch to coversin, a small recombinant C5-inhibiting lipocalin protein with the additional advantage of subcutaneous administration, hemolysis was halted and clinical symptoms improved significantly [57]. The AK585 study (ClinicalTrials.gov, NCT03427060) will further investigate if coversin may be a useful therapy for other PNH patients who are resistant to eculizumab therapy due to genetic variants of C5. Furthermore, C5 inhibition does not prevent the continuous extravascular clearance of C3 fragment-opsonized RBCs by macrophages in the reticulo-endothelial system, as first described by Risitano et al. [58]. This phenomenon results in low-level hemolysis persisting in the majority of PNH patients treated with eculizumab [59]. Susceptibility to this phenomenon has been linked to CR1, which can play a protective role against C3b opsonization. CR1 has two co-dominant alleles, which lead to high (H) or low (L) expression of CR1 on the RBC surface. This decreased expression on the RBC surfaces leads to reduced C3b decay, which increases opsonization levels that induce extravascular hemolysis. Thus, patients with an H/L or L/L genotype have been shown to require more transfusions upon eculizumab treatment than patients with the H/H genotype [60]. This ongoing extravascular hemolysis may be overcome by more upstream complement inhibition, at the C3 level, as discussed in more detail subsequently [61].

### Complement in AIHA

AIHA is a heterogeneous disease caused by autoantibody-initiated destruction of RBCs, in which complement activation may play a role. It is a rare disease with an incidence of 10–30 cases per million individuals in adults and with an even lower incidence in children [62]. AIHA can be classified as primary (idiopathic) or secondary. The latter relates to underlying diseases or conditions which trigger the humoral immune response, including lymphoproliferative malignancies, autoimmune diseases, infections, immunodeficiencies, and certain drugs [63, 64]. The diagnosis of AIHA is based on the presence of hemolytic anemia and the serological detection of anti-RBC autoantibodies or complement on the RBCs by the direct antiglobulin test (DAT). Autoantibodies in AIHA can be of IgG, IgA, and IgM isotypes. According to the thermal amplitude, defined as the highest temperature at which the autoantibody can bind its antigen, and the isotype, AIHA can be divided into warm (60–70% of cases) and cold (20–25% of cases) AIHA [65]. Cold AIHA (cAIHA) is further classified as primary cold agglutinin disease (CAD), typically associated with a low-grade lymphoproliferative disorder producing a (often low level) monoclonal IgM, while cold agglutinin syndrome (CAS) is secondary to an underlying disease [65, 66]. Less often, mixed or atypical forms of AIHA and paroxysmal cold hemoglobinuria (PCH) are diagnosed [63]. The degree of complement deposition on the RBC depends on the isotype and even IgG subclass, thermal amplitude, the recognized targets, and the number of bound autoantibodies. Autoantibodies from IgM isotype (and to a lesser extent, the IgG isotype subclasses 1 and 3) are strong complement activators and able to bind C1q upon RBC targeting. Thus, these are responsible for the activation of the CP of the complement system [67]. IgM autoantibodies are typically cold agglutinins associated with cAIHA, while warm IgM autoantibodies are rare.

Warm AIHA (wAIHA) is characterized by polyclonal autoantibodies with an optimal binding temperature at 37°C. The DAT is typically positive for autoantibodies of IgG and/or to a lesser extent of IgA class (15–20%). The density of warm autoantibodies of IgG or IgA type on the RBC membrane is usually not sufficient to serve as a binding place of C1q, and hemolysis in wAIHA is typically considered to be complement independent. Despite that, complement deposition, mainly C3d, may be detected in the DAT. We previously showed that in these cases, IgM isotype autoantibodies are often present and found indications that these are responsible for C3 deposition [62, 68]. In wAIHA, the opsonized RBCs are mostly cleared by FcR, but C3 deposition may add to the level of extravascular hemolysis in the spleen and liver (Fig. 2B) [66]. Intravascular hemolysis is not often seen in wAIHA.

In patients with cAIHA, the DAT is positive for complement, and IgM binding may also be detected. However, as IgM often has low affinity at body temperature, it may bind at the extremities or body parts with low temperature and detach from the RBC membrane at 37°C. Therefore, the detection of C3 fragments in the DAT may be the only remnant of the initial complement activity caused by an IgM autoantibody [68]. The involved IgM autoantibodies optimally bind to the RBC antigens at 3–4°C. They are likely only pathogenic if the thermal amplitude exceeds 30°C. Clinically, cAIHA is not only characterized by hemolytic anemia but also by RBC agglutination in the peripheral circulation, leading to acrocyanosis [69]. Although in patients with all types of AIHA, membrane-bound complement regulators are normally expressed; in severe cases, complement activation can result in the formation of the MAC, leading to intravascular hemolysis [61, 69–72].

While the majority of AIHA is either wAIHA or cAIHA, there are some rare variants. Mixed AIHA (10% of cases) is defined as the combination of a warm type of IgG autoantibodies and evidence of a cold type of autoantibody. The DAT is positive for IgG and C3d [65]. PCH is a very rare AIHA subtype. It occurs almost exclusively in young children in reaction to a viral infection and, although it can be severe, is a self-limiting disease. In adults, it is exceedingly rare and can be associated with infections (tertiary syphilis). PCH is caused by a strongly complement-activating polyclonal IgG autoantibody predominantly directed against the P antigen on the RBC. The IgG autoantibody binds only at low temperatures but is able to initiate complement activation to such an extent that it leads to the formation of the MAC and subsequently intravascular hemolysis [66]. The DAT is usually negative for IgG and frequently only positive for C3 fragments [73]. Finally, (rare) atypical forms of AIHA caused by warm IgM autoantibodies and AIHAs with a negative DAT exist [63, 65].

### Current management of AIHA

The AIHA subtype directs the choice of therapy. In secondary AIHA, treatment of the underlying disease is an important part of the therapy. Primary wAIHA is traditionally treated with corticosteroids, which leads to a response in approximately 80% of patients [63, 64]. However, many of these responses are temporary. Rituximab, an anti-CD20 antibody, is often considered as a second line of treatment with an overall response rate of around 80% [64, 65], although relapses are frequently observed [65]. In refractory or relapsed patients, splenectomy and alternative immunosuppressive drugs (such as azathioprine, mycofenolate mofetil, cyclosporin, and endoxan) can be considered [4, 64, 65]. The use of complement inhibitors has not been extensively studied in wAIHA thus far [74]. However, as complement activation may be part of the pathophysiology of wAIHA, complement inhibition with pegcetacoplan (C3 inhibitor) is investigated in an ongoing clinical trial. Also, a clinical trial with the C1q inhibitor ANX005 is planned including wAIHA patients with evidence of complement involvement (ClinicalTrials.gov, NCT03226678 and NCT04691570).

In primary cAIHA or CAD, the anemia is often mild to moderate, and many patients can be managed conservatively with the advice to avoid cold exposure. Some patients, however, suffer from significant hemolytic anemia or acrocyanosis despite thermal protection. In these cases, rituximab is the first line of treatment; however, the overall response rate is only 50%, and the median duration of response is less than 12 months [65, 66]. Cytotoxic combinations such as rituximab with bendamustine or fludarabine induce higher response rates (70–75%) and more sustained remissions (several years). However, these therapies are associated with short-term (infections, cytopenia) and long-term (secondary malignancies, stem cell toxicity) adverse events. In addition, time to response can be weeks to months, and over 25% of patients with CAD do not respond to chemotherapy [75, 76].

Recently, complement inhibition has become the focus of clinical studies in cAIHA. The first study of complement inhibition in CAD was a phase 2 prospective clinical trial with the C5 inhibitor eculizumab (DECADE) [77]. In twelve patients with chronic CAD, and one with acute CAS, eculizumab significantly reduced intravascular hemolysis and transfusion requirements. However, it did not normalize Hb levels and did not improve quality of life [77]. The lack of effect of this terminal complement inhibitor is probably related to ongoing extravascular hemolysis via phagocytosis in the liver. To date, the use of eculizumab has been discussed for the treatment of rare cases of severe intravascular hemolysis in exacerbations or refractory cAIHA [78].

Considering most of the hemolysis in cAIHA takes place via phagocytosis of C3b-opsonized RBCs in the liver, inhibition at a more proximal level of the complement cascade may be a more effective approach. Sutimlimab, a humanized C1s monoclonal antibody, was studied in phase 3 clinical trial for transfusion-dependent cAIHA patients (CARDINAL study, ClinicalTrials.gov, NCT03347396). It induced a rapid and sustained effect in patients with CAD with a resolution of hemolysis and a resolution of transfusion independency in 70% of the patients, together with an improvement in quality of life. These clinical improvements correlated with the normalization of complement factor C4 levels and decreased CP activity [79]. Results are awaited for the CADENZA study, where sutimilimab is investigated in the (more prevalent) transfusion-independent CAD population (ClinicalTrials.gov, NCT03347422). BIVV020, a monoclonal antibody targeting activated C1s, is currently studied in a phase 1b clinical trial. An ongoing phase 2 trial investigates the C3a/b inhibitor pegcetacoplan in CAD (ClinicalTrials.gov, NCT03226678). In line with upstream complement inhibition at the C1 level, the use of plasma-derived C1-INH, since long approved and available for the treatment of hereditary angioedema, has been reported in a single patient with cAIHA. Here, C1-INH was effective and safe in controlling complement-mediated AIHA [80, 81]. Currently, we are performing a phase 2 open-label study that examines the effect of co-administration of C1-INH and blood transfusion in severe cAIHA on the recovery of allogeneic RBCs (ClinicalTrialsRegister.eu: 2012-003710-13).

PCH is often transient, and treatment for PCH is mainly supportive of maintaining warm temperatures and blood transfusions if required [66, 73, 82]. As the hemolytic anemia in PCH is fully complement mediated, there is a rationale for the use of complement inhibitors in severe cases of PCH. There are some case reports describing the effect of eculizumab on intravascular hemolysis in patients with PCH with varying results [83, 84].

While there are currently no approved complement-directed therapies for cAIHA, complement inhibitors are expected to change the therapeutic landscape for this disease. But despite these promising results, it is important to realize that complement inhibition does not halt autoantibody production, and its binding to RBC epitopes as the underlying antibody-producing clone is not targeted. This is especially relevant in IgM-mediated AIHA with both symptomatic hemolysis and agglutination disease, as complement inhibition only targets the first but not the latter. Thus, complement inhibition at any level in the complement cascade does not benefit the patient suffering from acrocyanosis [77]. In these cases, a clone-directed approach, that is, with immunochemotherapy or possibly novel B cell-directed therapies such as BTK inhibition, may be more suited.

## Complement inhibition nowadays and in the future

### Disadvantages of the use of complement inhibitors

The development of terminal complement inhibitors has revolutionized the treatment of PNH, and the arrival of more proximal complement inhibitors potentially will have a dramatic impact on the treatment of complement-mediated AIHA. However, these treatments come with several limitations. First, as complement inhibition does neither cures nor affects the underlying disease, continuous treatment is needed to maintain response. In the case of eculizumab, this involves lifelong intravenous infusions at a bimonthly frequency, coming with immense expenses, limited patient compliance, and impairment of patients’ quality of life [45]. Especially in PNH, it is essential to strictly adhere to dosing schemes as any delay in infusion risks loss of C5 inhibition with subsequent massive hemolysis and secondary life-threatening (thrombotic) events. The increased half-life of ravulizumab in this respect overcomes some of these logistic aspects. Similarly, in cAIHA, the second-generation C1s inhibitor BIVV020 is expected to have a prolonged half-life compared to sutimlimab due to selectively inhibiting the activated form of C1s rather than total C1s. Currently, this agent is being evaluated in an extended phase 1b study (ClinicalTrials.gov, NCT04269551).

Second, complement is an important part of the host defense mechanism, especially against virulent encapsulated bacteria. Chronic inhibition warrants proper antimicrobial protection by a comprehensive vaccination schedule, while some also advise long-term antibiotics [85]. So far, real-world data concerning the use of eculizumab demonstrated an annual incidence of meningococcal disease of 0.5% per year. Importantly, 95% of meningococcal infections originated in patients not vaccinated against all serotypes. However, even with complete serotype vaccination, ex vivo data have shown increased baseline risks due to an eculizumab-induced inhibition of the pro-inflammatory peptide C5a, which is essential for upregulation of microbial phagocytosis [76]. Theoretically, the risk of such infections in patients treated with a more upstream inhibitor specific for C3, thereby both blocking bacterial opsonization and bacterial killing via MAC, may be even higher. Substantial clinical data are currently lacking, although small ongoing trials so far supported a favorable safety profile [86, 87].

Third, complement inhibition upstream of C5 may optimize achievement of treatment goals in the patients with ongoing hemolysis due to C3 deposition or for patients with C5 variation, rendering C5-targeted therapy ineffective [56, 57]. Finally, but only theoretically thus far, based on the function of the CP and the role of the C1 complex in immune complex clearance, inhibition of the proximal part of the CP could increase the risk of symptoms found in immune complex diseases, as is observed in patients with congenital CP deficiencies (C1q, C1r, C1s, C2, and C4) [88]. To date, there are no clinical data that support this hypothesis, but this should be closely monitored.

**Fig 3:**
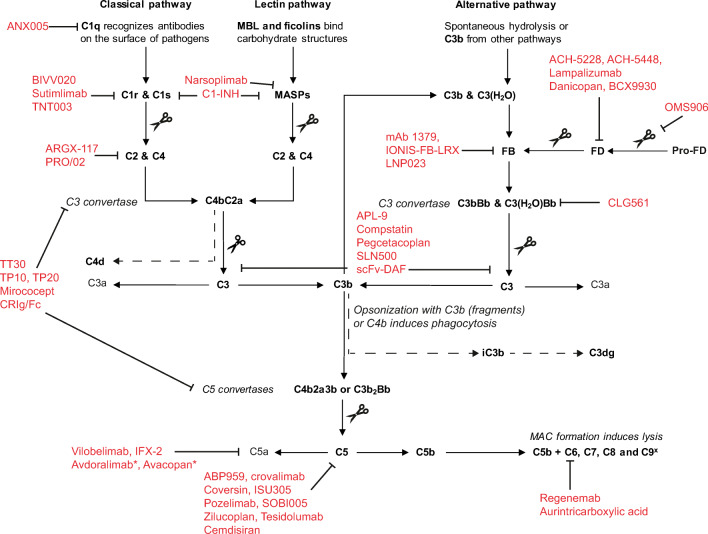
The complement system and targets of novel complement therapeutics. This overview shows the current drug developing landscape for AIHA and PNH. Scissors indicate the cleavage of a protein by a protease. Dotted lines indicate the breakdown of proteins. Figure adapted from [135]. *These drugs do not directly target C5a but rather the C5a receptor C5aR

Altogether, these arguments have nourished the search for additional therapeutics, which may target complement inhibition upstream of C5 at a target that is specific for the disease, facilitate more convenient non-intravenous routes of administration, and/or facilitate prolonged dose intervals due to increased half-life. To conclude this review, we will discuss novel potential targets, provide an overview of the complement therapeutics currently in the pipeline, and discuss their potential for the treatment of AIHA and PNH (for a full overview of agents in the pipeline, see Table 2 and Fig. 3).

**Table 2:** Overview of the complement therapeutics currently in the pipeline that could potentially aid in the treatment of AIHA and PNH

Target	Therapeutic	Type	Administration^2^	Target diseases	Phase	Other remarks	Company [refs]
C1r, C1s, MASP2	C1-INH	Recombinant or plasma-derived protein	IV	Hereditary angioedema	Approved	C1-INH was also reported to be successful in a patient with secondary AIHA	Alexion [80, 89–91]
MASP2	Narsoplimab (OMS271)	Monoclonal antibody	IV and SC	Atypical hemolytic uremic syndrome, IgA nephropathy, COVID-19	III	Narsoplimab targets the LP by inhibiting MASP2. Although aHUS is an AP-mediated disease, increased MASP-2 levels have been reported in aHUS, and inhibiting MASP2 could reduce endothelial cell damage in vitro.	Omeros [92–94]
C1s	BIVV020	Monoclonal antibody	IV	AIHA	I		Sanofi, NCT04269551
	Sutimlimab (BIVV009)	Monoclonal antibody	IV	AIHA (CAD)	III	A humanized variant of TNT003. First results of the phase III trial show rapid and sustained effects in preventing hemolysis, increasing hemoglobin levels, and improving quality of life.	Sanofi [79, 86, 87]
	TNT003	Monoclonal antibody	n.s.	AIHA (CAD)	Preclinical	Effective in inhibiting C3 deposition in plasma of CAD patients in vitro	Sanofi (Bioverativ, True North Therapeutics) [95]
C1q	ANX005	Monoclonal antibody	IV	Huntington disease, amythrophic lateral sclerosis, Guillan–Barre syndrome	II		Annexon [96]
C2	ARGX-117	Monoclonal antibody	IV	n.s.	Preclinical		Argenx [97]
	PRO-02	Monoclonal antibody	n.s.	Ischemia-reperfusion injury or antibody-mediated disease	Preclinical		Prothix [98]
C3	APL-9	Peptide	IV	Severe COVID-19	I/II		Apellis, NCT04402060
	Compstatin (AMY101, Cp40)	Peptide	SC or IV	Gingivitis; COVID-19	II		Amyndas, [83, 85, 86]
	Pegcetacoplan (APL-2)	Peptide	SC	AIHA; PNH; age-related macular degeneration	II (AIHA) and III		Apellis [99]
	SLN500	RNAi	n.s.	n.s.	Preclinical		Silence Therapeutics
	scFv-DAF	CD55 and antibody fragment fusion protein	n.s.	Myasthenia gravis	Preclinical	This fusion protein is designed to treat autoantibody-mediated disease	[100, 101]
C3 and C5 convertase	TT30 (ALXN1102, ALXN1103)	FH and CR2 fusion protein	IV and SC	PNH	I		Alexion [102, 103]
	TP10 and TP20	Recombinant sCR1	IV	Ischemia/reperfusion injury, cardiopulmonary bypass, vascular injury	II	TP20 is a modified version of TP10, using sLe^x^ groups to target it to the endothelium	Celldex [104–107]
	Mirococept (APT070)	Engineered recombinant sCR1	IV	C3 glomerulopathy, kidney transplantation	II	Phase II clinical trial in kidney transplantations underway	MRC [108–111]
	CRIg-Fc	CRIg and Fc fusion protein	n.s.	C3 glomerulopathy, experimental arthritis, ischemia/reperfusion injury	Preclinical	Fusion protein designed to bind C3 and inhibiting C3 and C5 convertases of the AP	Genentech [26, 112, 113]
C5	ABP 959	Monoclonal antibody	IV	PNH	II and III		Amgen [114]
	Eculizumab	Monoclonal antibody	IV	PNH, aHUS	Approved		Alexion [49, 50, 58, 77, 115]
	Ravulizumab	Monoclonal antibody	IV	PNH, aHUS	Approved		Alexion [55]
	Crovalimab (SKY59)	Monoclonal antibody	IV and SC	PNH	II	Antibody optimized for SC administration by sequential monoclonal antibody recycling technology and increased solubility	Roche [116]
	Coversin (nomacopan, rVA576)	Small molecule inhibitor	SC	PNH	II	Coversin can also bind mutated C5 found in a subgroup of Asian PNH patients, unlike eculizumab	Akari Therapeutics [57]
	ISU305	Monoclonal antibody	IV	PNH, aHUS	I		ACTRN-12619000694112
	Pozelimab (REGN3918)	Monoclonal antibody	IV and SC	CHAPLE^3^; PNH	II/III (CHAPLE); I (PNH)		Regeneron [117]
	SOBI005	Protein	n.s.	n.s.	Preclinical		Biovitrum
	Zilucoplan and Zilucoplan-XR	Peptide	SC	PNH; myasthenia gravis	II; III		UCB [118]
	Tesidolumab (LFG316)	Monoclonal antibody	IVT or IV	Age-related macular degeneration; PNH	II		Novartis, NCT02534909,NCT01527500
	Cemdisiran (ALN-CC5)	RNAi	SC	PNH; aHUS	II		Alnylam [119]
C5a	Vilobelimab (IFX-1; CaCP29)	Monoclonal antibody	IV	Hidradenitis suppurativa; ANCA-associated vasculitis; severe COVID-19 pneumonia; pyoderma gangrenosum; granulomatosis with polyangitis; microscopic polyangiitis; sepsis; systemic inflammatory response syndrome	II; III for COVID-19 pneumonia		InflaRx [120]
	IFX-2	Monoclonal antibody	IV	n.s.	Preclinical		InflaRX
C5aR	Avdoralimab	Monoclonal antibody	IV	COVID-19; bullous pemphigoid; advanced solid and hematological tumors	II		Innate Pharma, NCT04371367,NCT04563923,NCT04333914
	Avacopan	Small molecular antagonist	Oral	aHUS; ANCA-associated vasculitis	II; III		ChemoCentryx [121, 122]
C6	Regenemab (CP010)	Monoclonal antibody	n.s.	n.s.	Preclinical		Regenescance
C9	Aurintricarboxylic acid	Small molecule inhibitor	Oral	n.s.	Preclinical		[123, 124]
Factor B	mAb 1379	Monoclonal antibody	n.s.	n.s.	Preclinical		Taligen Therapeutics [125]
	IONIS-FB-LRX	Antisense RNA inhibitor	SC	Age-related macular degeneration; IgA nephropathy	II		Ionis NCT04014335
	LNP023	Small molecule inhibitor	Oral	PNH; IgA nephropathy; C3 glomerulopathy	III		Novartis [126]
Factor D	ACH-5228 (ALXN2050)	Small molecule inhibitor	Oral	PNH	II		Achillion/Alexion NCT04170023
	ACH-5448	Small molecule inhibitor	Oral	PNH	Preclinical		Achillion
	Lampalizumab	Monoclonal antibody (Fab)	ITV	Geographic atrophy/age-related macular degeneration	III		Genentech [127]
	Danicopan (ACH-4471)	Small molecule inhibitor	Oral	PNH	II	Danicopan is currently studied in a phase III trial as an add-on to C5 inhibition in order to prevent extravascular hemolysis (ALPHA study)	Achillion/Alexion [128]
	BCX9930	Small molecule inhibitor	Oral	PNH	I/II		BioCryst NCT04170023
MASP3	OMS906	Monoclonal antibody	IV	PNH	Preclinical	Inhibits the AP as MASP3 is the protein responsible for cleavage of pro-factor D into FD.	Omeros
Factor H	Anti-FH.07	Potentiating monoclonal antibody	n.s.	aHUS	Preclinical		Sanquin [129, 130]
	AMY201 (Mini FH)	Recombinant protein	n.s.	Age-related macular degeneration; PNH	Preclinical		Amyndas [131, 132]
	FH, FHmoss	Recombinant	n.s.	aHUS	Preclinical	Has not been tested in animal models for aHUS	Greenovation Biotech [133, 134]
Properdin	CLG561	Monoclonal antibody (Fab)	ITV	Age-related macular degeneration	I		Novartis, NCT01835015

1.Information on ongoing clinical trials obtained from ClinicalTrials.gov on 3 Dec 2020. If a therapeutic has not been published yet, the ClinicalTrials.gov identifier is provided

2.Abbreviations for administration routes: lV, intravenous; ITV, intravitreal; SC, subcutaneous

3.CHAPLE: CD55 deficiency with hyper-activation of complement, angiopathic thrombosis, and severe protein-losing enteropathy

### Developments in the complement field

In order to reduce extravascular hemolysis, an obvious direction for drug discovery lies in targeting the complement system at the level of C3. By targeting at this level, the opsonization of RBCs and subsequent phagocytosis can be prevented. Targeting complement at the C3 level has a clear benefit in that it allows for the targeting of all different activation pathways simultaneously, as these come together at the C3 level. Thus, complete inhibition of complement activity would be possible, if desired [136]. The main therapeutic candidates for the inhibition of C3 are compstatin and its analogs. Compstatin is a cyclic tridecapeptide, originally found in a random peptide library upon screening for C3b binding, which showed to have direct C3 inhibiting properties [137]. Ever since, several analogs of compstatin have been developed in order to optimize production, pharmacokinetics, and delivery of the drug [136, 138]. Compstatin analog Cp40 has been shown to prevent hemolysis and opsonization of PNH RBCs and inhibition of intravascular and extravascular hemolysis in AIHA serum in vitro [139, 140]. Up to date, pegcetacoplan (formerly APL-2), a pegylated compstatin analog suitable for subcutaneous administration, has been deemed safe in phase I clinical trial. Currently, a phase II trial is on its way for the usage of pegcetacoplan in AIHA (ClinicalTrials.gov: NCT03226678), as well as a phase III trial for PNH (ClinicalTrials.gov: NCT04085601). The subcutaneous administration likely implicates a significant reduction in patient burden. However, it may come at the cost of infectious complications, as C3 blocking both halts opsonization and terminal complement activation. C3 deficient patients suffer from recurring bacterial infections, but to what extent this corresponds to a pharmacologically induced C3 deficiency remains to be determined [141].

There are certain therapeutics in development that target the CP and/or LP specifically. Since in AIHA the CP is the main route of complement activation, blocking the CP is an appealing alternative to blocking C5. The first drug used to target the CP/LP is recombinant or plasma-purified C1-INH, which is approved for usage in hereditary and acquired angioedema, where patients suffer from C1-INH deficiency [142]. As described previously, C1-INH was successful in a patient with refractory AIHA based on a single-case report [80]. Currently, a phase II clinical study is underway in AIHA patients (ClinicalTrialsRegister.eu: 2012-003710-13/NL). The CP can also be targeted at other levels, using sutimlimab and BIVV020 to inhibit C1s and thus prevent C2 cleavage as described previously, or by using an mAb against C2 directly [86, 87, 97]. C2 especially is a promising target to block the CP and LP, as it has the lowest concentration of the CP and LP components and would therefore potentially require lower and/or less frequent dosage of inhibitors [8]. A recent study described ARGX-117 as an inhibiting mAb against C2, which successfully inhibited CP activity in cynomolgus monkeys [97].

Next to specific targeting of the CP, several therapeutics are in development to specifically inhibit the AP. This seems especially of interest for PNH, which is mainly a disorder of the AP. A benefit of targeting the AP is that an infection can still be cleared with the help of the complement system via the LP or CP, thus potentially reducing infection risk [143]. Furthermore, C3b opsonization could potentially be slowed down by inhibiting the AP, which would reduce extravascular hemolysis. Interestingly, there are a few therapeutics in the pipeline that can be administered orally and target FB, FD, or properdin. FD can be targeted in two ways: either by inhibiting FD directly or by inhibiting MASP3, which cleaves pro-factor D into FD. Properdin stabilizes the AP C3 convertase and thereby extends its activity [8]. Conversely, inhibiting properdin does not completely block C3 convertase formation but rather shortens its activity [144]. Inhibition of the AP as a mechanism of action has been demonstrated to be effective in animal models of antibody-induced arthritis and membranous nephropathy, and several clinical trials in PNH are currently in progress [126]. Especially, LNP023 (ClinicalTrials.gov: NCT03439839), ACH-5228 (ClinicalTrials.gov: NCT03439839), danicopan (ClinicalTrials.gov: NCT04170023), and BCX9930 (ClinicalTrials.gov: NCT04469645) are of particular interest as these can be administered orally (Table 2). If successful, these oral administration routes could open up novel possibilities of complement-targeting treatment with a limited patient burden.

Another way of inhibiting complement activity while minimizing the infection risk is by utilizing mechanisms of complement regulators, which guard against dysregulated complement activation. Several recombinant proteins that mimic (parts of) complement regulators are in development [141]. An example is TT30, a FH and CR2 fusion protein designed to inhibit the AP at sites of complement activation. The CR2 domains bind to the C3b fragments at sites of complement activation, where the FH domains can then fulfill their regulatory function [102]. In vitro, TT30 inhibited complement-mediated hemolysis and C3b opsonization of PNH RBCs, while in vivo studies in monkeys have shown almost complete inhibition of the AP and partial inhibition of the CP upon subcutaneous TT30 administration [102, 103]. A phase I trial for TT30 in PNH however was recently terminated, as the enrollment criteria could not be met (ClinicalTrials.gov, NCT01335165). Another interesting approach is the potentiation or suppletion of fluid-phase complement regulators, such as FH and FI, which could seriously reduce AP activity and thus aid in the reduction of both intravascular and extravascular hemolysis [135]. An interesting example is a potentiating antibody, anti-FH.07, that has been developed for FH [129]. As this is an AP regulator, potentiating FH would mainly be of interest in PNH. Anti-FH.07 has already been shown to be able to enhance the function of mutated FH in vitro on RBCs [130].

Finally, several novel treatments that target the terminal complement pathway, similar to eculizumab and ravulizumab, are in development. Although their clinical effect and application are probably similar to that of the currently approved therapeutics, these new therapeutics are designed to simplify the administration, which will increase convenience and compliance to therapy and may reduce costs. Coversin, crovalimab, zilucoplan, and cemdisiran (all in phase II trials) are all C5 inhibitors that can be administered subcutaneously [57, 116, 118, 119]. Lastly, aurintricarboxylic acid is a C9 small molecule inhibitor that can be taken orally and was effective in inhibiting hemolysis of PNH RBCs in vitro [123, 145].

## Concluding remarks

The success stories of eculizumab and ravulizumab have shown that there is a need for drugs that specifically target activated complement components that can be subcutaneously or orally administered in diseases in which the RBC is a target of the complement system. For almost all complement components, there is currently a specific drug available or in development, which will have an immense impact on the therapeutic approach to rare complement-mediated diseases, including those of the RBC such as PNH and AIHA. Optimizing complement-targeting therapies in terms of safety and convenience will further improve therapeutic options and will have an immense impact on the therapeutic approach of rare complement-mediated hemolysis.

## Abbreviations

AP: alternative pathway
AIHA: autoimmune hemolytic anemia
CAD: cold agglutinin disease
CAS: cold agglutinin syndrome
CP: classical pathway
DAT: direct antiglobulin test
FB: factor B
FH: factor H
FI: factor I
LP: lectin pathway
MAC: membrane attack complex
PCH: paroxysmal cold hemoglobinuria
PIGA: phosphatidylinositol glycan-A
PNH: paroxysmal nocturnal hemoglobinuria
RBC: red blood cell
